# Integrated transcriptomics and metabolomics decipher differences in the resistance of pedunculate oak to the herbivore *Tortrix viridana* L.

**DOI:** 10.1186/1471-2164-14-737

**Published:** 2013-10-28

**Authors:** Birgit Kersten, Andrea Ghirardo, Jörg-Peter Schnitzler, Basem Kanawati, Philippe Schmitt-Kopplin, Matthias Fladung, Hilke Schroeder

**Affiliations:** 1 Thünen Institute of Forest Genetics, Sieker Landstrasse 2, D-22927, Grosshansdorf, Germany; 2 Helmholtz Zentrum München, Institute of Biochemical Plant Pathology, Research Unit Environmental Simulation, Ingolstädter Landstr. 1, D-85764, Neuherberg, Germany; 3 Helmholtz Zentrum München, Research Unit Biogeochemistry and Analytics, Ingolstädter Landstr. 1, D-85764, Neuherberg, Germany

**Keywords:** RNA sequencing, Transcriptomics, Metabolomics, Defence response, *Quercus robur*, *Tortrix viridana*

## Abstract

**Background:**

The interaction between insect pests and their host plants is a never-ending race of evolutionary adaption. Plants have developed an armament against insect herbivore attacks, and attackers continuously learn how to address it. Using a combined transcriptomic and metabolomic approach, we investigated the molecular and biochemical differences between *Quercus robur* L. trees that resisted (defined as resistant oak type) or were susceptible (defined as susceptible oak type) to infestation by the major oak pest, *Tortrix viridana* L.

**Results:**

Next generation RNA sequencing revealed hundreds of genes that exhibited constitutive and/or inducible differential expression in the resistant oak compared to the susceptible oak. Distinct differences were found in the transcript levels and the metabolic content with regard to tannins, flavonoids, and terpenoids, which are compounds involved in the defence against insect pests. The results of our transcriptomic and metabolomic analyses are in agreement with those of a previous study in which we showed that female moths prefer susceptible oaks due to their specific profile of herbivore-induced volatiles. These data therefore define two oak genotypes that clearly differ on the transcriptomic and metabolomic levels, as reflected by their specific defensive compound profiles.

**Conclusions:**

We conclude that the resistant oak type seem to prefer a strategy of constitutive defence responses in contrast to more induced defence responses of the susceptible oaks triggered by feeding. These results pave the way for the development of biomarkers for an early determination of potentially green oak leaf roller-resistant genotypes in natural pedunculate oak populations in Europe.

## Background

Plants suffer constantly from herbivore pressure, and their defence responses are therefore highly evolved and tightly regulated. With more than 200,000 secondary metabolites, plants directly resist microbial and insect attacks, animal predation [1,2], and multiple environmental stresses [3,4]. The suite of secondary metabolites in plants is composed both of metabolites that are constitutively expressed in different plant tissues and of inducible compounds that complete the overall armament of plants in response to herbivore feeding [5,6].

To ensure optimal defence responses, plants must be able to up- and down-regulate primary and secondary metabolic pathways at every level to exert temporal and spatial control in an effective and efficient way, thereby minimising damage and ensuring vegetative growth and reproduction [7]. For this purpose, plants have evolved constitutive and induced defence mechanisms. Preformed molecular [8], chemical, and physical mechanisms may reduce the accessibility or availability of the plant resources to herbivorous insects. For example, one of the most important groups of constitutive defence compounds that act against herbivores and pathogens are the condensed tannins (proanthocyanidins; PA) [7,9]. These are polyphenolic compounds synthesised via the flavonoid biosynthetic pathway. Analyses of specific PAs have been performed in many tree species, such as poplar and oaks (e.g., [10,11]) and in herbaceous plants (e.g., [9]). In oak foliage, for example, different levels of condensed tannin content in combination with nitrogen content seem to be essential in determining the susceptibility to herbivorous insects [12,13]. In addition to the condensed tannins, the so-called hydrolysable tannins (i.e., gallotannins, ellagitannins) are also important and have only recently become a focus of research into the defence response [14]. Interestingly, insect specialists prefer lower tannin content than do insect generalists [9].

Inducible defence reactions involve a broad range of molecules whose synthesis is temporally controlled [7]. We know of at least two types of inducible defence responses: the direct defences that inhibit the growth or development of herbivorous insects and the indirect defences that include the plant volatiles, which may, for example, attract the parasitoids and predators of the herbivore [15,16].

Transcript profiling using DNA microarrays has significantly improved our understanding of the regulatory and transcriptional networks of gene activation/inactivation in plants during plant-insect interaction [17-19]. However, while this technology is restricted to profiling transcripts that are represented by corresponding DNA probes on the microarray, recent developments in RNA sequencing (RNAseq) allow the genome-wide profiling and quantification of transcripts, and these approaches can be used to study plant defence responses in more comprehensive detail [20-22]. To date, the use of RNAseq in studies of herbivory has been rare. Gilardoni *et al.*[23] analysed the *Nicotiana attenuata* transcriptome using SuperSAGE and 454 sequencing after elicitation with fatty acid-amino acid conjugates known to act as elicitors in *Manduca sexta* herbivory.

As transient or constitutive end products of the cascade that begins with gene activation, the constituents of the metabolome define the biochemical phenotype of an organism. Thus, quantitative and qualitative measurements of the plant metabolome during herbivory can provide a wide overview of the biochemical status of the plant and essential information regarding the influence of metabolite levels on the phenotype [24].

In the present work, we applied RNAseq and non-targeted metabolome analysis, performed using Fourier Transform Ion Cyclotron Mass Spectrometry (FT-ICR-MS) [25], to examine the transcriptional and metabolomic differences in pedunculate oak (*Quercus robur* L.) varieties that differ in their degree of defoliation and susceptibility to herbivory by the green oak leaf roller (*Tortrix viridana* L., Lepidoptera: Tortricidae). *T. viridana* is a specialist herbivorous insect that feeds only on species of the genus *Quercus*[26,27]. In Central Europe, the perpetual outbreak of the green oak leaf roller is one reason for oak decline events reported during the last century. During a past outbreak episode in Germany in the years 2003–2005, in which *T. viridana* caused almost the complete defoliation of oaks in a selected forest stand in North Rhine-Westphalia, we observed that a few individual oaks were remarkably less defoliated than neighbouring trees. We defined these less-defoliated individuals as resistant ('T-oaks’) and the heavily defoliated trees as susceptible ('S-oaks’) [28]. In recent work, we demonstrated that the resistance of T-oaks to herbivore attack by *T. viridana* is related to the amount and scent of herbivory-induced plant volatiles (HIPVs). In the same study, we showed that the T- and S-oaks differed in their polyphenolic leaf constituents [29].

To unravel the underlying molecular mechanisms related to the resistance and susceptibility of oaks towards herbivory by *T. viridana*, we performed controlled laboratory experiments to identify candidate genes that exhibited induced differences in their expression patterns after insects feeding. Moreover, analysing the unfed control plants aided in the identification of candidate genes that exhibit constitutive expression differences between the oak types. To complete our systems biological approach, we comparatively analysed the metabolome of T- and S-oaks to correlate gene expression patterns and metabolite profiles. Moreover, this analysis provided the opportunity to identify the overall metabolomic differences between T- and S-oaks in addition to the local and systemic changes induced by *T. viridana* feeding or by developmental alterations in plant metabolite patterns.

## Results

### Transcriptional differences between T- and S-oaks after *T. viridana* feeding

As a first step, we used the MapMan tool [30] for displaying the transcriptional differences between T- and S-oaks after 16 h of *T. viridana* feeding to obtain a global overview of the related cellular pathways. All transcripts showing any difference in their expression level (RPKM-value: reads per kilobase of exon model per Million mapped reads) between the T- and S-oaks after *T. viridana* feeding were included in this analysis. When comparing the two oak types, 30 MapMan functional categories (BINs) showed a significantly different average BIN response (p < 0.05, Wilcoxon rank sum test in the MapMan tool; Additional file 1) compared to the response of all other BINs. The most significant of these BINs are related to photosynthesis and ribosomal protein synthesis, while other differences were identified in BINs related to chromatin structure, redox, targeting to mitochondria, and other cellular functions (Additional file 2).

**Figure 1:**
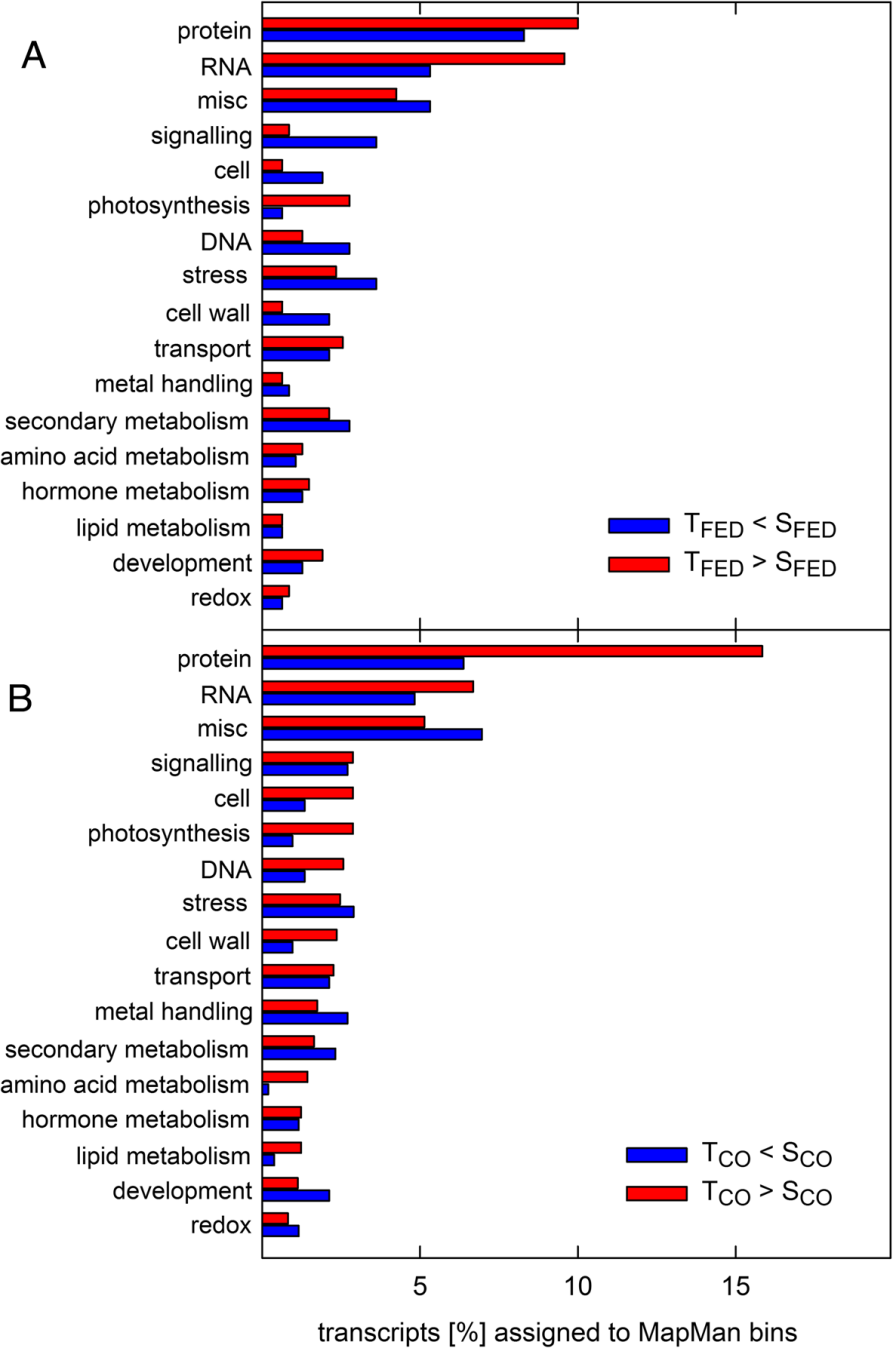
**Functional composition of the different candidate transcript groups.** Transcripts from the T_FED_ > S_FED_- (red) and T_FED_ < S_FED_- (blue) groups **(A)** and T_CO_ > S_CO_- (red) and T_CO_ < S_CO_- (blue) groups **(B)** were assigned to MapMan BINs and transcripts per BIN were counted (counts in% of total transcript counts in the candidate group on the X-axes). Only MapMan BINs showing at least 3 members in all groups were displayed. Unassigned transcripts were not displayed. CO, control sample; FED, fed sample.

In the second step, we selected candidate transcripts that were potentially involved in the different transcriptional responses of T- and S-oaks to *T. viridana* feeding. In total, we found 858 transcripts that were differentially expressed in response to *T. viridana* feeding. Of these, 389 had higher expression values in T-oaks than in S-oaks (T_FED_ > S_FED_-group; log2 fold change ≥ 1.5), while 469 had lower expression values (T_FED_ < S_FED_-group; log2 fold change ≤ -1.5; Additional file 3). Figure 1A depicts the distributions of these transcript groups (T_FED_ > S_FED_ and T_FED_ < S_FED_) with regard to BINs. A strikingly higher percentage of transcripts of the T_FED_ < S_FED_-group were present in the BINs related to signalling, cell, DNA, stress, and cell wall formation compared with the T_FED_ > S_FED_-group. The BINs RNA and photosynthesis showed the opposite trend (Figure 1A).

**Figure 2:**
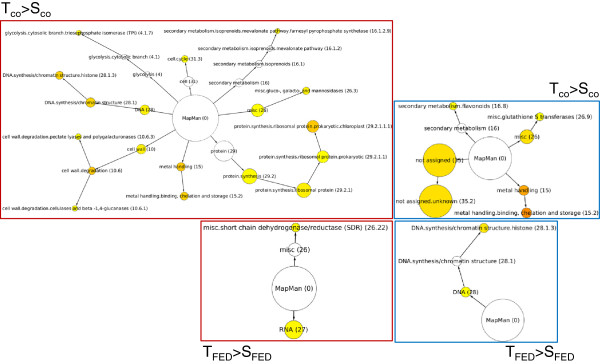
**Functional enrichment analysis in the different candidate transcript groups.** Over-representation of specific MapMan BINs was tested in the indicated transcript groups, in comparison to the *Q. robur* transcript reference set. Coloured nodes go from yellow to dark orange, representing p-values from 5E-2 to 5E-7. White notes represent MapMan BINs that are not significantly over-represented in the group. The size of a node is proportional to the number of transcripts annotated to that node.

Both transcript groups (T_FED_ > S_FED_ and T_FED_ < S_FED_) were further analysed for a statistical over-representation of specific BINs compared to the *Q. robur* reference set that was used for transcript mapping. In the T_FED_ > S_FED_-group, the RNA synthesis and short chain dehydrogenase/reductase BINs were significantly over-represented compared to the reference set (Figure 2). In contrast, the DNA BIN and the chromatin structure-related histone BIN were over-represented in the T_FED_ < S_FED_-group (Figure 2).

### Constitutive transcriptional differences between T- and S-oaks

To elucidate the potential differences between the constitutive transcript profiles of T- and S-oaks, we compared the transcript expression values between unfed T- and S-oak control samples. Although these samples represent pooled samples of different S- and T-oak individuals, one has to consider that some of the differentially expressed genes identified from this comparison between S- and T-oak controls would contribute to other phenotypic differences than resistance to the green oak leaf roller.

Considering all transcripts with any difference in the values between the samples, 28 BINs were identified that showed expression differences that deviated from those of all other BINs (Additional file 1). Among these BINs were the E3 (E3 ubiquitin ligase) BIN and the flavonoids BIN, which are related to secondary metabolism (Additional file 2).

In total, 1,464 transcripts showed constitutively different expression levels. Of these, 955 transcripts had higher expression levels in T-oaks compared to S-oaks (T_CO_ > S_CO_; log2 fold change ≥ 1.5), while 509 transcripts had lower expression levels (T_CO_ < S_CO_; log2 fold change ≤ -1.5; Additional file 3). It is obvious at first glance that a much higher percentage of the T_CO_ > S_CO_-group transcripts (red bars) were present in the protein BIN compared with those of the T_CO_ < S_CO_-group (blue bars; Figure 1B). The same trend in distribution was also observed for the cell, photosynthesis, DNA, cell wall, amino acid metabolism, and lipid metabolism BINs (Figure 1B). It is interesting to note that the cell, DNA, and cell wall BINs showed an inverse profile of transcript enrichment in the insect-fed leaves (Figure 1A).

When we analysed the enrichment of specific BINs in the T_CO_ > S_CO_-group compared to the *Q. robur* reference set, we observed that several BINs showed significant over-representation (Figure 2); among these were many protein synthesis-related BINs. With regard to secondary metabolism, the farnesyl pyrophosphate synthetase BIN of the cytosolic isoprenoid pathway was also over-represented in this group. Two BINs related to cell wall degradation, were also over-represented in the T_CO_ > S_CO_-group: (i) the pectate lyases and polygalacturonases BIN and (ii) the cellulases and beta-1,4-glucanases BIN (Figure 2). In the T_CO_ < S_CO_-group, transcripts corresponding to glutathione-S-transferases and metal handling (especially metal binding, chelation, and storage) showed an over-representation. With regard to secondary metabolism, we observed a significant enrichment of transcripts related to flavonoid backbone biosynthesis in this group (p < 0.05; Figure 2).

Table 1 (T_CO_ > S_CO_) and Table 2 (T_CO_ < S_CO_) summarise the 10 most differentially expressed transcripts in each group (Related sequences in Additional file 4). We observed dramatically higher expression levels in the T-controls than in the S-controls (log2 fold change > 6.5) for transcripts weakly similar to *Arabidopsis thaliana* transcripts encoding *PDF1* (protodermal factor 1; 71% maximum amino acid identity), a protein phosphatase 2C family protein (70% maximum amino acid identity), and a *GDSL*-motif lipase/hydrolase family protein (50% maximum amino acid identity; Table 1). Lower expression levels in T-controls compared to S-controls (log2 fold change < -6.5) were detected for transcripts moderately similar to *A. thaliana* transcripts encoding the ubiquitin extension protein 1 (*ERD16*, Early Response to Dehydration 1; 99% maximum amino acid identity) and osmotin 34 (69% maximum amino acid identity; Table 2).

**Table 1:** Top10-transcripts of the Tco > Sco-group with known functional MapMan annotation

**Identifier**	**MapMan sub-bin**	**Putative function according Mercator (score of the blast hit in italics)**	**RPKM T**_**CO**_	**RPKM S**_**CO**_	**Log2 fold T**_**CO**_**/ S**_**CO**_
WZ0AQRAP12YP16FM1	development. unspecified	weakly similar (*129*) to AT2G42840 PDF1 (PROTODERMAL FACTOR 1)	11.5	0.0	9.4
WZ0AQRAP12YM18FM1	protein. post-translational modification	weakly similar to (*182*) AT4G38520 protein phosphatase 2C family protein / PP2C family protein	31.3	0.1	8,5
Gnl|UG|Qro#S57156573	misc. GDSL-motif lipase	weakly similar (*180*) to AT4G18970 GDSL-motif lipase/ hydrolase family protein	3.7	0.0	6.8
Gnl|UG|Qro#S57132810	misc. protease inhibitor/ seed storage/ lipid transfer protein (LTP) family protein	weakly similar (*170*) to AT2G45180 protease inhibitor/seed storage/lipid transfer protein (LTP) family protein weakly similar (*145*) to 14KD_DAUCA 14 kDa proline-rich protein DC2.15 precursor - *Daucus carota*	6.4	0.1	6.7
WZ0AQRAQ11YF07FM1	DNA. synthesis/ chromatin structure. histone	weakly similar (*159*) to AT5G59910 HTB4; DNA binding | weakly similar (*160*) to H2B_GOSHI Histone H2B - *Gossypium hirsutum*	27.4	0.3	6.6
Gnl|UG|Qro#S57098114	protein. degradation. serine protease	moderately similar (*392*) to AT4G17040 ATP-dependent Clp protease proteolytic subunit, putative weakly similar (*106*) to CLPP_CHLVU ATP-dependent Clp protease proteolytic subunit (EC3.4.21.92)	5.0	0.1	6.6
Gnl|UG|Qro#S57144797	RNA. regulation of transcription. MYB-related transcription factor family	weakly similar (*191*) to AT1G74840 myb family transcription factor	13.9	0.2	6.4
Gnl|UG|Qro#S57095514	cell wall. degradation. cellulases and beta -1, 4-glucanases	moderately similar (*314*) to AT1G70710 CEL1, ATGH9B1 (ARABIDOPSIS THALIANA GLYCOSYL HYDROLASE 9B1); cellulase/ hydrolase, hydrolysing O-glycosyl compounds	15.9	0.2	6.3
WZ0AQRAP12YB01FM1	RNA. processing. ribonucleases	weakly similar (*182*) to MAL11_MALDO Major allergen Mal d 1 (Mal d I) *- Malus domestica*	40.8	0.8	5.7
WZ0AQRAP10YM20FM1	stress. abiotic. unspecified	moderately similar to (*237*) AT3G05950; germin-like protein, putative	0.9	0.0	5.6

Related sequences and GenBank accession numbers are available in Additional file 4.

**Table 2:** Top10-transcripts of the Tco < Sco-group with known functional MapMan annotation

**Identifier**	**MapMan subbin**	**Putative function according Mercator (score of the blast hit in italics)**	**RPKM T**_**CO**_	**RPKM S**_**CO**_	**Log2 fold T**_**CO**_**/ S**_**CO**_
WZ0AQRAQ11YC24FM1	protein. degradation. ubiquitin	moderately similar (*257*) to AT3G52590 UBQ1 (UBIQUITIN EXTENSION PROTEIN 1), EMB2167, ERD16, HAP4 UBQ1; protein binding / structural constituent of ribosome weakly similar (*150*) to UBIQ_WHEAT Ubiquitin	0.2	396.7	-10.8
Gnl|UG|Qro#S57141407	stress. biotic	moderately similar (*226*) to AT4G11650 ATOSM34 (osmotin 34) moderately similar (*285*) to P21_SOYBN Protein P21 - *Glycine max*	7.4	1514.8	-7.7
Gnl|UG|Qro#S57077753	cell. organisation	moderately similar (*380*) to AT5G12380 annexin, putative moderately similar (*471*) to ANX4_FRAAN Annexin-like protein RJ4 - *Fragaria ananassa*	1.3	103.7	-6.3
Gnl|UG|Qro#S57131911	protein. synthesis. ribosomal protein. eukaryotic. 40S subunit.S8	moderately similar (*297*) to AT5G59240 40S ribosomal protein S8 (RPS8B) moderately similar (*313*) to RS8_MAIZE 40S ribosomal protein S8 - *Zea mays*	0.2	12.2	-5.7
Gnl|UG|Qro#S57149481	stress. abiotic. unspecified	moderately similar (*231*) to AT5G53160 unknown protein	0.1	2.5	-5.6
Gnl|UG|Qro#S57088372	stress. biotic. PR-proteins	weakly similar (*134*) to AT5G17680 disease resistance protein (TIR-NBS-LRR class), putative	0.7	13.7	-4.3
Gnl|UG|Qro#S57110966	misc. peroxidases	weakly similar (*191*) to AT5G51890 peroxidase weakly similar to (*188*) PER2_ARAHY Cationic peroxidase 2 precursor (EC 1.11.1.7; PNPC2) - *Arachis hypogaea*	0.1	1.0	-4.2
Gnl|UG|Qro#S57139400	transport. sugars	moderately similar (*241*) to AT4G36670 mannitol transporter, putative weakly similar (*109*) to HEX6_RICCO Hexose carrier protein HEX6 - *Ricinus communis*	0.1	2.4	-4.2
Gnl|UG|Qro#S57094988	secondary metabolism. isoprenoids. carotenoids. carotenoid cleavage dioxygenase	very weakly similar (*87.8*) to AT3G63520 CCD1 (CAROTENOID CLEAVAGE DIOXYGENASE 1), ATCCD1, ATNCED1, NCED1; 9-cis-epoxycarotenoid dioxygenase	0.3	5.8	-4.1
gnl|UG|Qro#S57087752	protein. post-translational modification	moderately similar to (*294*) AT3G51630 WNK5 (WITH NO LYSINE (K) KINASE 5), ZIK1, ATWNK5; protein kinase	1.7	28.1	-4.1

Related sequences and GenBank accession numbers are available in Additional file 4.

### Transcriptional responses induced by *T. viridana* feeding in T- and S-oaks

After comparing the transcript profiles of fed and unfed T- and S-oaks, we were interested in the transcriptional responses of T- and S-oaks that were induced by *T. viridana* feeding. As the expression values from the fed samples were derived from a different type of Solexa reads (36 bp single-end reads) than the expression values from the controls (101 bp single-end reads), this bioinformatic analysis has to be interpreted carefully. Nevertheless, we obtained a general overview of the cellular functions involved in the defence responses of *Q. robur* to *T. viridana* and identified additional differences between the oak types.

All transcripts exhibiting an increase or decrease in their expression value after feeding (log2 fold change > 0 or < 0), compared to the corresponding unfed controls were considered in a MapMan analysis. In total, 48 BINs showed significant different average BIN responses compared to the response of all other BINs in both T- and S-oaks (p < 0.05, Wilcoxon rank sum test in the MapMan tool; Additional files 1 and 2). Among these were BINS related to the light reaction of photosynthesis, to the synthesis of prokaryotic and eukaryotic ribosomal proteins and to abiotic stress (Additional file 2). Changes in chromatin structure, especially in the associated histones indicate an involvement of epigenetic transcriptional regulation in the host defence (Additional file 2).

BINs that exhibited significant differences only in T-oaks comprised, among others, those related to cell wall degradation, GDSL-motif lipases, and protein targeting to the secretory pathway (Additional file 2). In S-oaks, the BINs related to steroid synthesis, squalene metabolism, metal handling, E3 ubiquitin ligases, and redox regulation were among those with a significant different BIN response.

**Figure 3:**
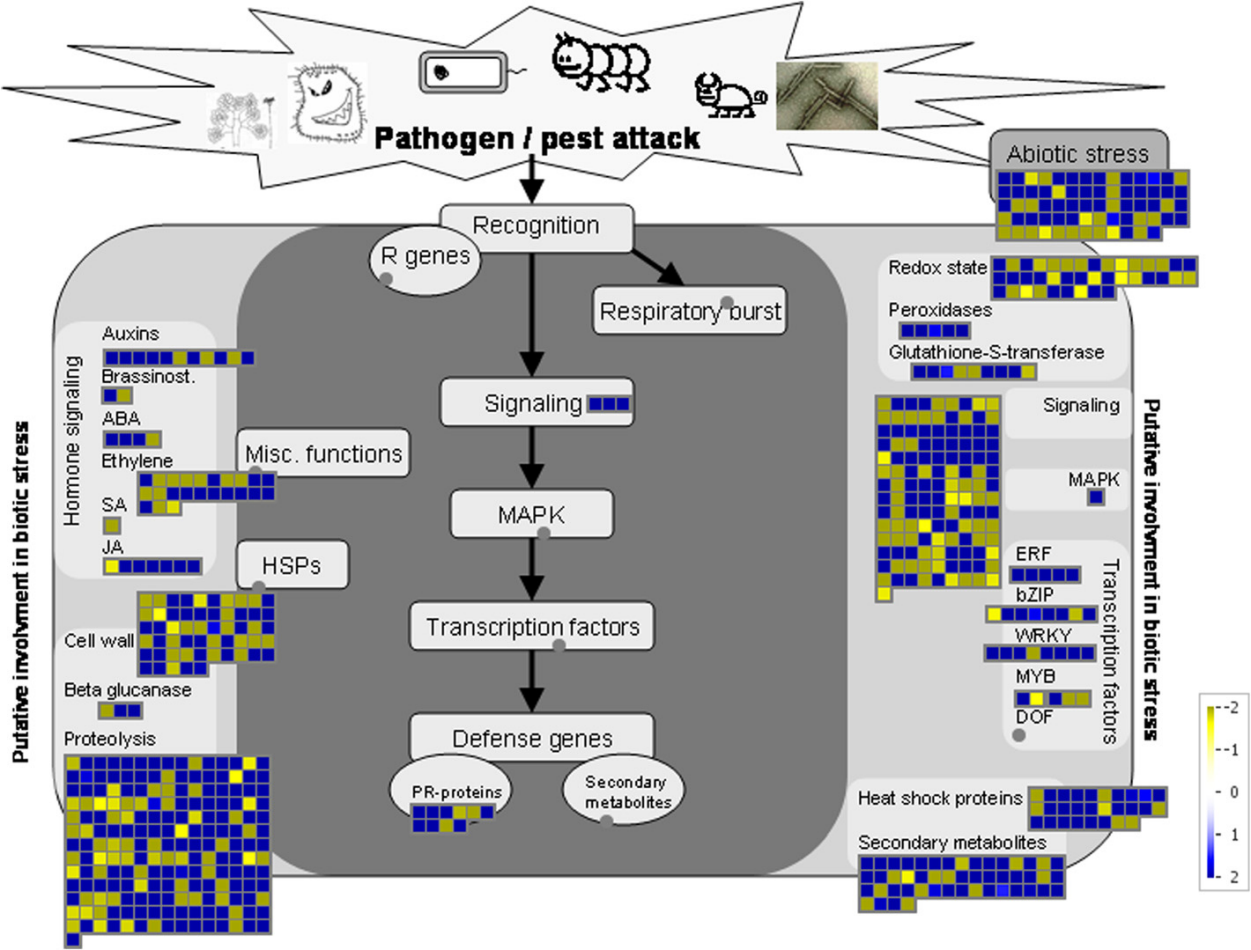
**Transcriptional changes induced by *T. viridana *feeding in both T- and S-oaks.** All transcripts induced by *T. viridana* feeding in both T- and S-oaks were mapped to the 'Biotic stress’ drawing in the MapMan tool [30]. Blue boxes, transcripts up-regulated after feeding in both T- and S-oaks (T_IND_ > = 1.5 and S_IND_ > = 1.5; mean value of T_IND_ and S_IND_ was mapped); yellow boxes, transcripts down-regulated after feeding in both T- and S-oaks (T_IND_ < = -1.5 and S_IND_ < = -1.5; mean value of T_IND_ and S_IND_ was mapped). Dots representing putative metabolites appear in grey as no related data were mapped.

We further identified groups of up- or down-regulated transcripts after *T. viridana* feeding by comparing the expression values between the different treatments. Considerably more transcripts showed an up-regulation (2,932 transcripts; log2 fold change ≥ 1.5) than showed a down-regulation (1,177 transcripts; log2 fold change ≤ -1.5) after *T. viridana* feeding in both T- and S-oaks (Additional file 3). The expression value changes (up- or down-regulation) that were induced by *T. viridana* feeding in both T- and S-oaks were mapped to the 'Biotic stress’ drawing in MapMan, which represents transcripts that may be involved in biotic stress (Figure 3). Most of the induced transcripts were assigned to BINS related to proteolysis, signalling, abiotic stress, cell wall, secondary metabolites, redox state, and heat shock protein. With regard to hormone signalling, transcripts assigned to ethylene, auxin, and jasmonate BINs were the most mapped transcripts. Most of the transcripts assigned to jasmonate, peroxidases, *ERF* (ethylene-responsive factors) and *WRKY* transcription factor BINs were up-regulated by *T. viridana* feeding in both T- and S-oaks (Figure 3).

### Transcripts expressing putative cell-wall-degrading enzymes

We observed an enrichment of transcripts encoding cell wall-degrading enzymes in the T_CO_ > S_CO_ group. In particular, the abundance of pectate lyase and polygalacturonase transcripts, in addition to cellulase and beta-1,4-glucanase gene transcripts, was increased (Figure 2). Thus, we became specifically interested in the differences in the expression of these transcripts between T- and S-oaks.

**Figure 4:**
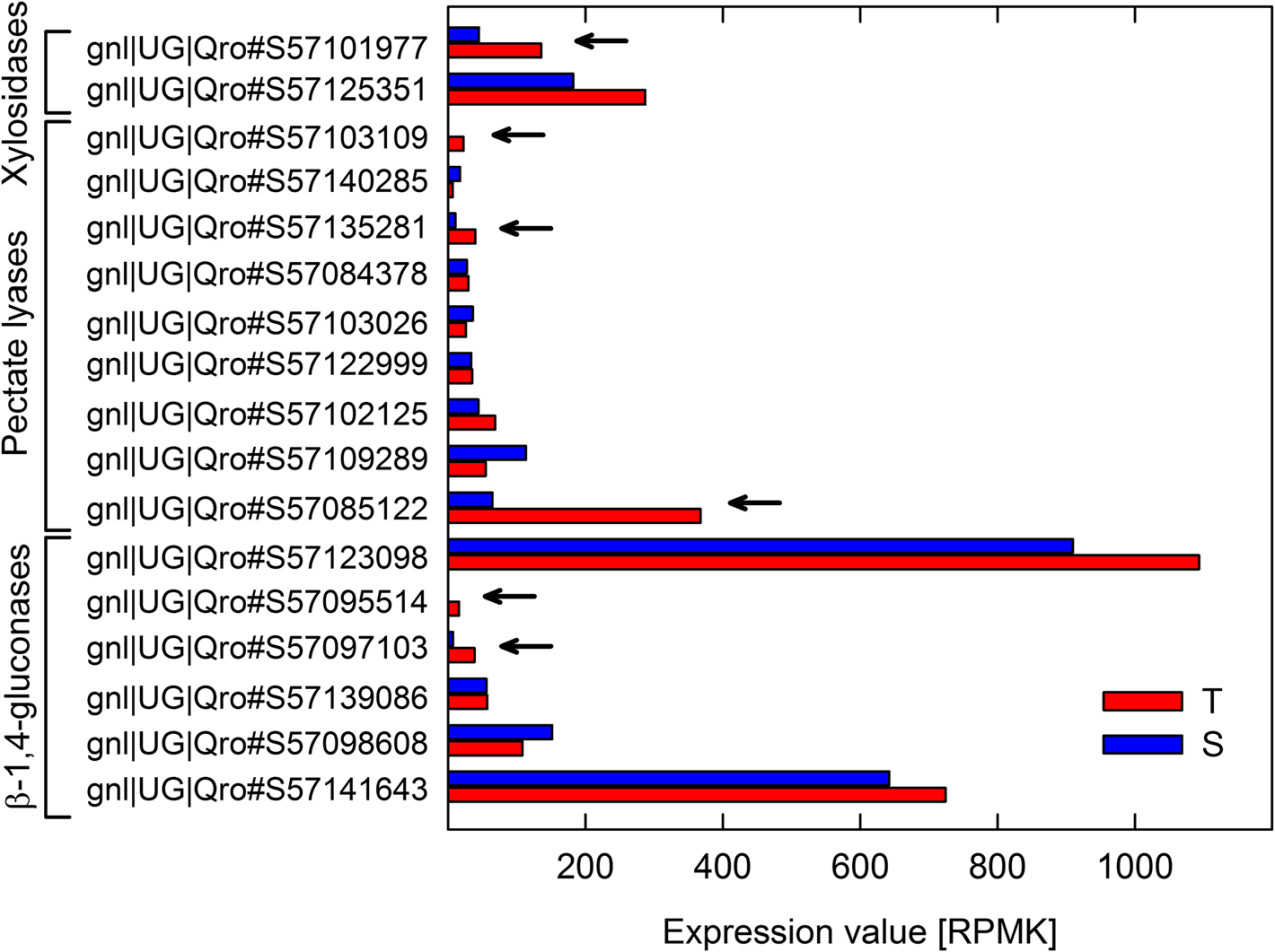
**Transcript levels of cell wall degrading enzymes in T- and S-oak controls.** Expression values (RPKM) of transcripts assigned by MapMan to the cell wall degradation BIN are presented for T-oak controls (red) and S-oak controls (blue). Only transcripts where the sum of RPKM values of T- and S-oak controls was at least 10 are presented. Arrows mark transcripts with log2 fold changes ≥ 1.5 when comparing expression values of T- with S-oak controls.

Figure 4 shows the expression values of all transcripts that were assigned to the cell wall degradation BIN and that exceeded a specific expression value in the T-oak controls (red bars) and S-oak controls (blue bars). Most of the transcripts showed higher expression values in T-oaks than in S-oaks (Figure 4; all transcripts with clearly higher expression in T-oaks compared to S-oaks were marked by a star; log2 fold change ≥ 1.5).

### Experimental validation of differential expression of candidate genes by PCR

**Figure 5:**
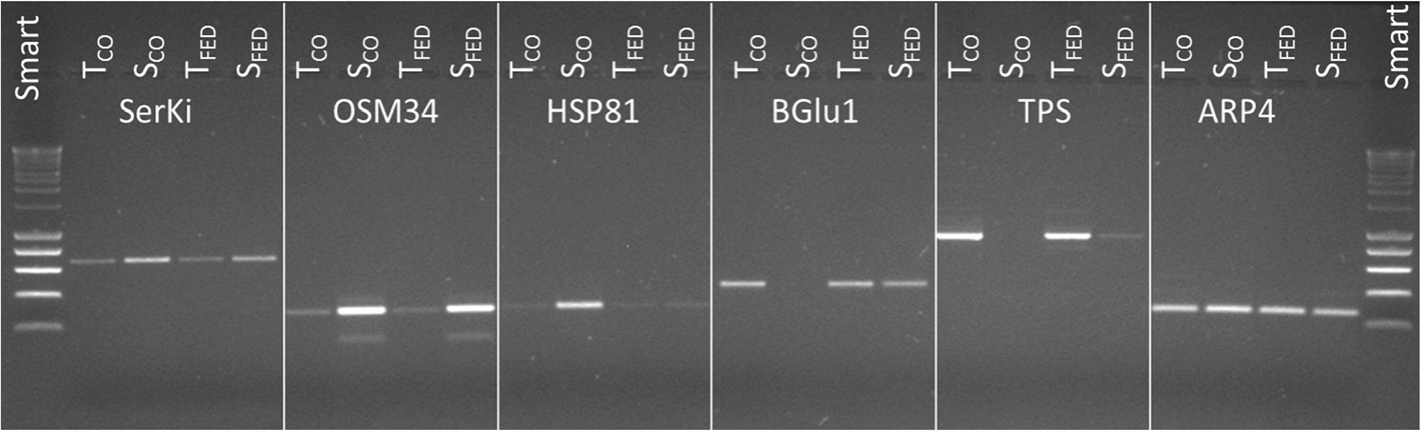
**Validation of candidate genes.** Agarose gel image of semi-quantitative PCR products of candidate genes. T_CO_, unfed control of T-oaks; S_CO_, unfed control of S-oaks; T_FED_, fed T-oaks; S_FED_, fed S-oaks; Smart, used size ladder. For expression values (RPKM) and used amount of DNA see Table 3.

Five genes with different expression levels for T- and S-oaks, namely, genes expressing a putative serine kinase, osmotin 34, HSP81 (a heat shock protein), *CEL1* (a beat-1,4-glucanase), a putative sesquiterpene synthase, and the housekeeping gene *ARP4* (encoding a putative actin-related protein), were chosen for a PCR-based validation of their expression (Table 3; related sequences in Additional file 4). The serine kinase (SerKi) showed a high constitutive expression value in S-oaks (S_CO_) and an equally strikingly low expression value in both fed (T_FED_) and control (T_CO_) T-oaks (Table 3, Figure 5). Osmotin 34 (OSM34) was chosen for its combination of an extremely high constitutive expression value in S_CO_, a high expression value in S_FED_, and low expression values in both fed (T_FED_) and control (T_CO_) T-oaks (Tables 2 and 3, Figure 5). The expression levels of HSP81 were also high in S-oaks (S_CO_ and S_FED_) and low in T-oaks (T_CO_ and T_FED;_ Table 3; Figure 5). To represent genes with a high constitutive expression value in T_CO_ and a slightly increased expression value in S_FED_, the beta-1,4-glucanase gene CEL1 (BGlu1) was used (Tables 1 and 3, Figure 5). Finally, a sesquiterpene synthase (TPS) showed very low constitutive expression values in S_CO_ but had high expression values in both T_CO_ and T_FED_ and slightly increased expression values in S_FED_ (Table 3, Figure 5).

**Table 3:** Expression values of candidate genes used for semi-quantitative PCR

**Identifier**	**Gene name**	**Expression values (RPKMs)**				**ng DNA for PCR**
		**T**_**CO**_	**S**_**CO**_	**T**_**FED**_	**S**_**FED**_	
gnl|UG|Qro#S57094678	SerKi	9	41	9	12	10
gnl|UG|Qro#S57141407	OSM34	7.4	1514.8	8.4	362.9	2
gnl|UG|Qro#S57133728	HSP81	1.1	16.6	2.1	6.7	10
gnl|UG|Qro#S57095514	BGlu1	15.9	0.2	2.78	3.6	6
Qr_TPS_putative_terpene synthase	TPS*	59.5	1.1	56.9	10.5	10
gnl|UG|Qro#S57081658	ARP4*	126.4	124.3	74.1	81.8	10

Related sequences and GenBank accession numbers are available in Additional file 4.

T_CO_, unfed control of T-oaks; S_CO_, unfed control of S-oaks; T_FED_, fed T-oaks; S_FED_, fed S-oaks. Asterisk at TPS and ARP4, mapping has been performed later than for the other candidates.

### Metabolomic differences between T- and S-oaks after *T. viridana* feeding

**Figure 6:**
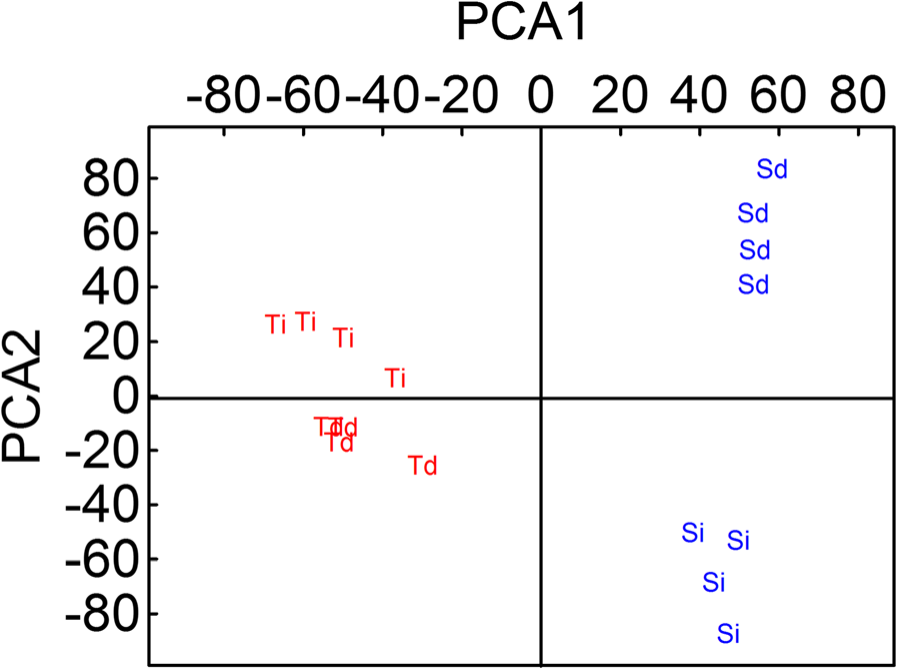
**Principle component analysis (PCA) of non-target metabolomics.** Metabolic differences between T- (red) and S-oaks (blue) from damaged (Td and Sd) and intact (Ti and Si) leaves. All plants were fed by *T.viridana* for 32 h and the leaves were 'old’ (6–8 weeks after budbreak) (PCA1 = 8% and PCA2 = 7% of total X-variable variation).

Principal components analysis (PCA) identified clear metabolic differences between T- and S-oaks at 32 h after the onset of feeding by *T. viridana* larvae (Figure 6) by explaining a total of 15% of the variance in metabolites differences. Furthermore, the distinct metabolic profiles of intact (I) and directly damaged (D) leaves showed that local and/or systemic defence responses were induced in the plant within 32 h of herbivore feeding.

**Figure 7:**
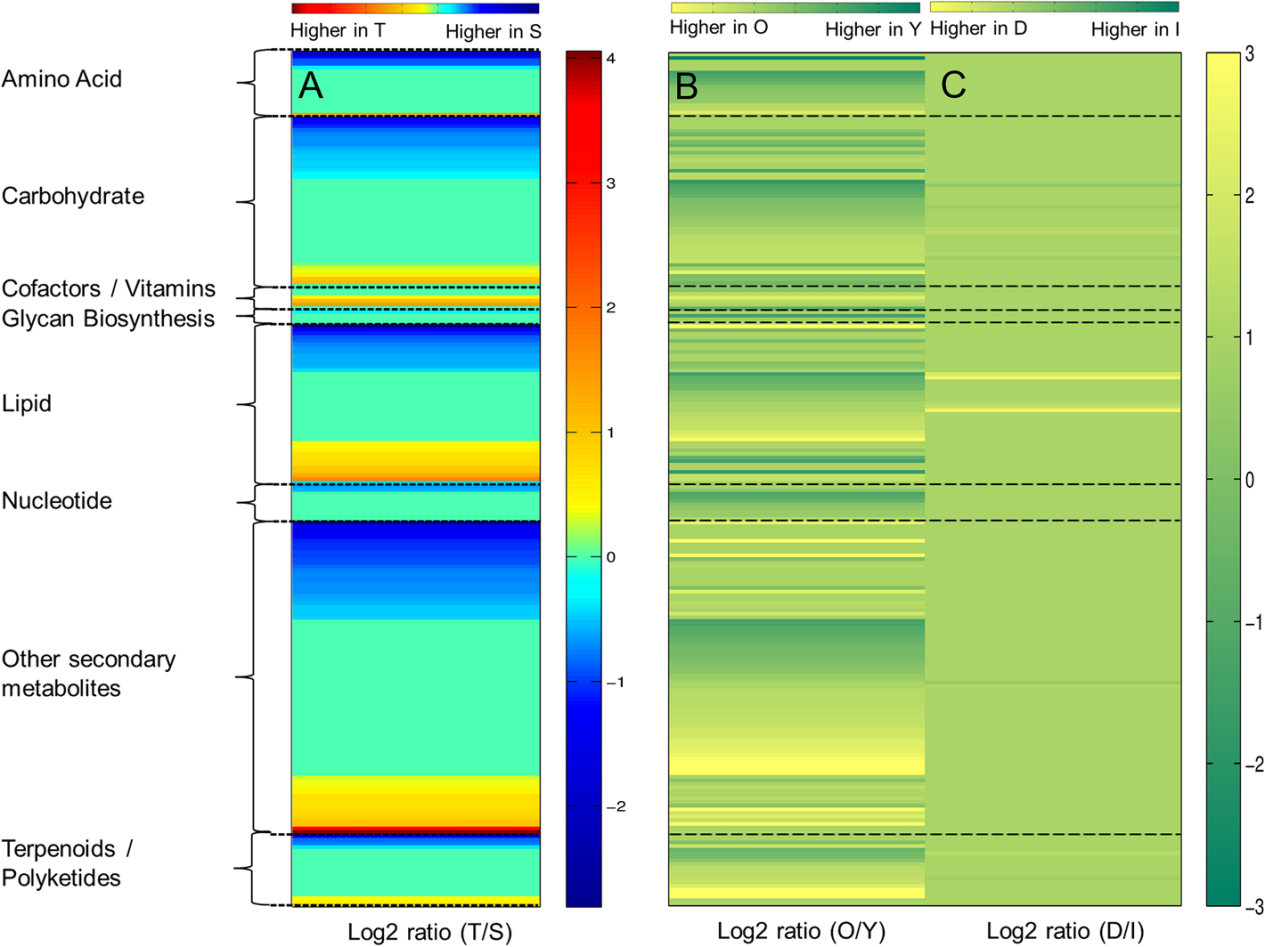
**Metabolic differences induced by *T. viridana *in T- and S-oaks, as response of phenotypes, leaf age dependences and systemic responses.** Heat maps of KEGG annotated significantly discriminant masses originated by partial least square regression (PLSR) analysis of metabolomic data showing **(A)** phenotypic/genotypic differences between T- and S-oaks, **(B)** leaf age developmental changes between fed plants (32 h) at early developmental leaf stage (2–4 weeks after budbreak; 'Y’) and 4 weeks later (6–8 weeks after budbreak 'O’), **(C)** differences between damaged leaf (d) and intact leaves (I) of fed plants. Metabolites are grouped into the main metabolism pathways according to KEGG [31]. Significance was tested with Martens’ test [83].

To gain insights into the compounds correlated with each group, we performed a discriminant partial least squares regression (PLSR) analysis (data not shown) and annotated the significant discriminant masses (Martens test) using the KEGG, LMPK, HMDB, and ChemSpider databases (Additional file 5). The identified metabolites showed a distinct metabolic accumulation that was characteristic of their metabolic pathway and cluster group (Figure 7A). In general, we found that 110 metabolites were either up- or down-regulated in the two different T- and S-oaks (Figure 7A, Additional file 5), which suggests that these metabolites might be good candidates for molecular biomarkers of the two T- and S-genotypes.

Each oak genotype displayed different levels of metabolites that could be grouped based on their KEGG classification [31] into metabolites belonging to amino acid, carbohydrate, cofactors, and vitamins, glycan, lipid, nucleotide, and secondary metabolism (terpenoid/ polyketide and other secondary metabolites, including alkaloids, flavones and flavonoids, and phenylpropanoid) classes (Figure 7A, Additional file 5). Among the metabolites showing strong differences in levels between T- and S-oaks, we focused our attention on the most abundant metabolites measured. Nicotinate ribonucleotide, an important precursor of nicotinamide adenine dinucleotide (NAD), was found to be strongly up-regulated in T-oaks. Several end-products of secondary metabolism, particularly galloylated flavonol glycosides (of which kaempferol galloylgalactoside and quercetin galloylglucoside were the most likely candidates among all potential isomers), were found to occur at levels that were 10- to 20-fold higher in T-oaks than in S-oaks. The amounts of these compounds did not differ between D and I leaves, which indicates that they likely show constitutive differences between T- and S-plants. The concentration of corilagin, a tannin and galloyl derivative, was higher in T-oaks. The amount of ellagic acid, another galloyl derivative typically found in oaks, was also greater in T-oaks. In addition, many biosynthetic precursors of condensed tannins were found at higher levels in T-oaks. Amongst these were flavan 3-ol derivatives, such as epigallocatechin, a catechin with an additional phenolic hydroxyl group. Additionally, some phenolic intermediates, such as coumaric acid, sinapoyl malate, coumaroyl quinic acid, were much more abundant in T-oaks than in S-oaks.

Conversely, S-oaks showed higher levels of basic flavonol glycosides. Luteolin glycoside, quercetin glycoside, and a methoxykaempferol glycoside were highly abundant in S-oak leaves (MS intensities > 10^7^) and also showed a greater relative difference between T- and S-oaks (log_2_ (T/S) < -1; Additional file 5). Additionally, free, unconjugated flavonols, such as luteolin and quercetin, showed relatively greater abundance in S-oaks than in T-oaks (Additional file 5, see also the next section).

In terms of lipid metabolism factors, the phospholipid glycerophosphoinositol was more abundant in T-oaks than in S-oaks. Conversely, the abundance of the glycerophosphodiester glycerophosphoglycerol was higher in S-oaks.

Globally, S-leaves showed higher levels of metabolites related to sugar metabolism, particularly the monosaccharides (e.g., rhamnose) and disaccharides (e.g., neohesperidose, sucrose), melibiitol from galactose metabolism, and different sugar intermediates (e.g., ribose 5-phosphate, xylose derivatives). T-oaks also contained higher amounts of other sugars, such as glucarate (an intermediate of ascorbate metabolism) and ribose 5-diphosphate, compared to S-oaks. Metabolites from nucleotide metabolism were significantly more abundant in S-oaks, which had relatively high levels of cyclic adenosine monophosphate (cAMP) and cytidine monophosphate.

We could generally characterise the developmental changes in leaf metabolism using 169 metabolites that exhibited significantly altered expression in our study. Leaf maturation was associated with an increase in amino acids related to phenylalanine derivatives, while it was also associated with decreased levels of tryptophan, aspartic acid, and homoserine derivatives (Figure 7B, Additional file 5). Young leaves were rich in sugars, whereas older leaves showed a large accumulation of fatty acids (FA) that are related to herbivore defence (e.g., hallactone). Leaf development was also characterised by strong differences of the levels of specific flavonoids and phenolic compounds.

Local and systemic responses were indicated by significant changes in the levels of 14 metabolites between D- and I-leaves. Directly damaged leaves exhibited a greater accumulation of FA, FA oxidation products related to wounding responses, signalling compounds, and healing agents, such as traumatic acid, tuberonic acid, tuberonic acid glucoside, linolenic acid, and 13-L-hydroperoxylinoleic acid. Conversely, intact leaves had relative higher levels of carbohydrates and secondary metabolites (Figure 7C, Additional file 5).

### Combined mapping of metabolites and transcripts to metabolic pathways

In an initial study [29], we described differences in the emission pattern of HIPVs among T- and S-oaks. T-oaks displayed higher emission rates of sesquiterpenes (α-farnesene and germacrene D), while the HIPV pattern of S-oaks was dominated by monoterpenes and the irregular acyclic homoterpene 4,8-dimethylnona-1,3,7-triene (DMNT), a derivative of the sesquiterpene nerolidol produced by oxidative degradation by a cytochrome P450 monooxygenase. Moreover, we found distinct differences in the phenolic compound composition of T- and S-oaks, and these differences were analysed in more detail, as described above. In the present analysis, we observed a significant enrichment of transcripts related to the biosynthesis of flavonoid backbones in the T_CO_ < S_CO_-group (Figure 2). Moreover, there were significant changes detected in the flavonoids BIN as well as the related chalcones BIN when comparing all transcriptional differences between the T- and S-oak controls in MapMan (Additional file 1). To gain deeper insights into the regulation of these two metabolic pathways in both oak genotypes, a combined mapping of transcriptomic and metabolomic data to these pathways was performed.

**Figure 8:**
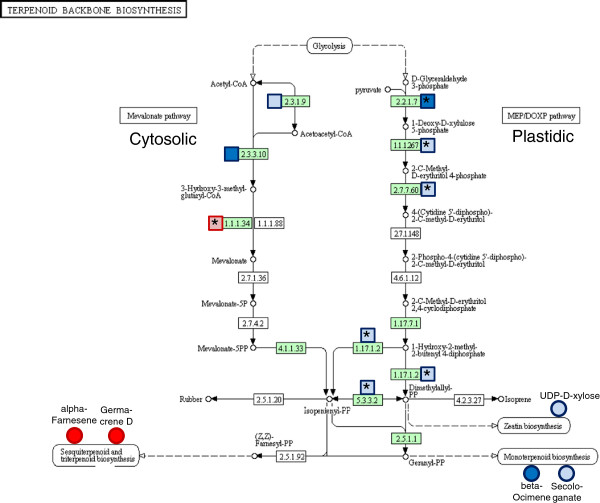
**Combined mapping of transcriptomic and metabolomic data onto the KEGG reference pathway “terpenoid backbone biosynthesis”.** Log2 fold changes of RPKM ratios according to Additional file 5 and of mass intensities according to Additional file 4 were mapped on the KEGG reference pathway. Enzymes highlighted in green are enzymes present in the *Q. robur* reference transcript set that was used for as a reference for quantification of the transcript data. Log2 fold changes of alpha-Farnesene, Germacrene D, and beta-Ocimene were taken from Ghirardo *et al.*[29]. Boxes/circles in light red, T_FED_ > S_FED_ (0.2 ≤ log2 fold change of RPKM/mass intensity ratio < 1.0); boxes/circles in dark red, T_FED_ > > S_FED_ (log2 fold change of RPKM/mass intensity ratio ≥ 1.0); boxes/circles in light blue, T_FED_ < S_FED_ (-1 < log2 fold change of RPKM/mass intensity ratio ≤ -0.2); boxes/circles in dark blue, T_FED_ < < S_FED_ (log2 fold change of RPKM/mass intensity ratio ≤ -1.0); big asterisk in the box, specific mapping of the transcript to the pathway “terpenoid backbone biosynthesis”; no asterisk in the box, unspecific mapping of the transcript to several pathways.

**Figure 9:**
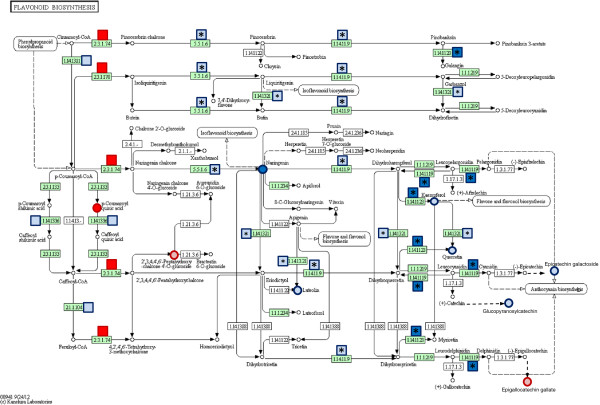
**Combined mapping of transcriptomic and metabolomics data onto the KEGG reference pathway “flavonoid biosynthesis”.** Log2 fold changes of RPKM ratios according to Additional file 6 and of mass intensities according to Additional file 5 were mapped on the KEGG reference pathway. Big asterisk in the box, specific mapping of the transcript to flavonoid biosynthesis; small asterisk in the box, mapping of the transcripts to the pathways “flavonoid biosynthesis” and “flavone and flavonol biosynthesis”; no asterisk in the box, unspecific mapping of the transcript to several pathways. For further information see legend of Figure 8.

Ratios (log2 fold changes) of transcript expression values and mass intensities were mapped to the KEGG pathways of terpenoid and flavonoid backbone biosynthesis (Figures 8 and 9). The data clearly show an increase in the transcript levels of the plastidic 2C-methyl-D-erythritol 4-phosphate (MEP) pathway for isoprenoid biosynthesis in S-oaks compared to T-oaks (Figure 8, right panel). Only one transcript mapped specifically to the mevalonate pathway (Figure 8, left panel, blue box representing EC 1.1.1.34) with higher transcript levels in T-oaks compared to S-oaks. The other two transcripts that mapped, i.e., acetyl-CoA C-acetyltransferase (EC 2.3.1.9.) and hydroxymethylglutaryl-CoA synthase (EC 2.3.3.10), are known to be involved in several KEGG pathways.

Figure 9 summarizes the transcriptomic and metabolomic data with regard to flavonoid backbone biosynthesis. Most of the transcripts and metabolites showed higher levels in S-oaks compared to T-oaks, which indicates an increase in the biosynthesis of basic flavonoid compounds in S-oaks after *T. viridana* feeding. Only chalcone synthase (EC 2.3.1.74; EC 2.3.1.170), the first enzyme in the flavonoid pathway, was expressed at a higher level in T-oaks than in S-oaks (red boxes in Figure 9). The transcripts from this gene did not specifically map to flavonoid biosynthesis. Therefore, the specific contribution of the expressed transcript to flavonoid backbone biosynthesis cannot be deduced from the transcript data.

## Discussion

In the present study, we aimed to discover the underlying genetic and metabolic basis for the differing susceptibilities of T- and S-oaks to *T. viridana* feeding.

**Figure 10:**
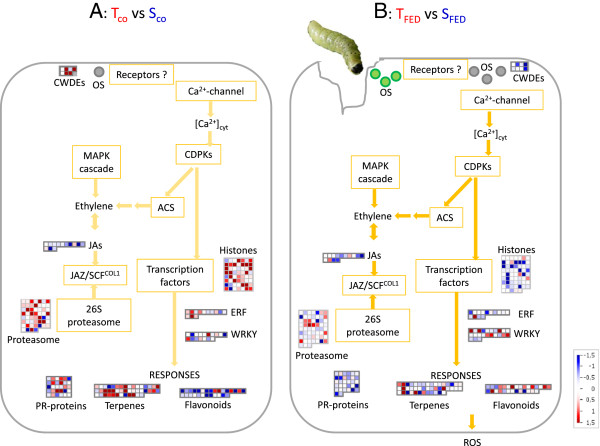
**Model of a signalling cascade for oak’s constitutive and induced defence response.** The model of the cascade is derived from a model recently published by Arimura *et al.*[32]. **A:** In the unfed control, the cascade is expected to be triggered by some 'damaged-self’ oligosaccharids (OS; grey circles) acting as elicitors activated by constitutively expressed cell wall degrading enzymes (CWDE; higher expressed in T-oaks than in S-oaks). **B:** Feeding by the leaf chewing insect *T. viridana* induces the release of herbivore-derived OS (green circles; elicitors) as well as of 'damaged-self’ OS and therefore initiates the cascade. The cascade itself is the same for the constitutive and induced defence response with different expression of transcripts in T- and S-oaks. Red squares represent transcripts stronger expressed in T-oaks and blue squares represent transcripts with higher expression in S-oaks. Transcripts assigned to the following MapMan BINs are presented: cellulases and beta -1,4-glucanases (CWDEs) belonging to cell wall degradation, jasmonate (JAs) related to hormone metabolism, proteasome (Proteasome) belonging to protein degradation, isoprenoids (Terpenes) and flavonoids (Flavonoids) related to secondary metabolism, ERF transcription factor family (ERF, ethylene-responsive factors), WRKY transcription factors (WRKY) belonging to regulation of transcription, histone (Histone) related to DNA synthesis/chromatin structure. Abbreviations: ACS, 1-aminocyclopropane-1-carboxylate; JAZ, jasmonate ZIM-domain; OS, oligosaccharids (elicitors); ROS, reactive oxygen species; SCF, SCF-type E3 ubiquitin ligase SCF^COI1^.

Plant defence responses to herbivory are driven by both herbivore-induced factors (e.g., elicitors, effectors, wounding) and plant signalling (e.g., phytohormones and plant volatiles; Figure 10) [32]. Figure 10 summarizes the constitutive and induced transcriptomic and metabolomic differences in T- and S-oaks responding to green oak leaf roller herbivory. The transcript levels of cell wall degrading enzymes (CWDE) are constitutively high in T-oaks (Figure 10A) but were found to be more inducible in S-oaks (Figure 10B). Changes in hormone signalling are likely to occur via the CDPK (Ca^2+^-dependent protein kinases) and MAPK (mitogen-activated protein kinase) cascades. Moreover, transcriptional changes at transcription factor genes are most likely responsible for the eventual activation of several defence response genes, such as those involved in the synthesis of volatiles and pathogen-related genes (Figure 10). The activated cascade results in a different response in T- and S-oaks mainly characterised by transcriptomic and metabolomic differences in the biosynthesis of tannins, flavonoids and terpenes (which is discussed in detail below).

### The cell wall as the first barrier for invading herbivores

The plant cell wall is the first line of defence against invading pathogens and herbivores. Plants have evolved sensory mechanisms to detect pathogens and herbivores, including the indirect sensing of the impact of the invader on the host cell wall ('damaged self’) [33,34]. In the present comparison, we discovered higher transcript levels of plant CWDEs (polygalacturonases and beta-1, 4-glucanases) in T-oak controls (Figure 10A), including increased levels of transcripts for the putative cellulase Cel1 (Figures 2 and 4; Table 1). At first glance, it is striking that T-oaks constitutively express higher levels of transcripts encoding enzymes that are normally inhibited by plants, e.g., by secreting polygalacturonase-inhibiting proteins, when being attacked by the polygalacturonases of a pathogen [35]. Conversely, cell wall-degrading enzymes may activate defence responses by releasing oligosaccharides as elicitors. Thus, the increased expression of several beta-1,4-glucanase genes, such as CEL1, in T-oaks (Tables 1 and 3; Figures 4, 5 and 10A) may result in higher constitutive levels of oligosaccharides. Additionally, cellulose-derived oligosaccharides have been shown to act as elicitors [36]. So far, there are no reports on the influence of higher constitutive expression levels of plant beta-1,4-glucanases and polygalacturonases on pathogen resistance. However, it was shown that tobacco and Arabidopsis plants expressing a fungal polygalacturonase are more resistant to microbial pathogens and have constitutively activated defence responses [37].

### Oxidative burst, protein phosphorylation signalling and hormone signalling

Beyond the cell wall, the response cascade continues within the cell membrane (Figure 10). Oral secretions from herbivores can initiate plant cell trans-membrane potential (Vm) depolarization, an early response to herbivore feeding that is associated with the opening of voltage-dependent Ca^2+^ channels, changes in the intracellular Ca^2+^ concentration and the generation of reactive oxygen species (ROS), such as H_2_O_2_ (Figure 10B) [32,38]. Immediately after this event, protein phosphorylation signalling via mitogen-activated protein kinase (MAPK) cascades, as well as via calcium-dependent kinases (CDPKs), triggers the production of phytohormones. After *T. viridana* feeding, we primarily observed the induction of transcripts associated with ethylene, jasmonic acid, and auxin production in both T- and S-oaks (Figures 3, 10). Genes related to jasmonic acid (JA) formation, for example, are already constitutively expressed at high levels (Figure 10A) and exhibit greater induction in S-oaks than in T-oaks (Figure 10B).

### Transcriptional differences in transcription factor genes and histone genes

Hormone signalling may also trigger transcriptional changes at transcription factor genes (Figure 10), which in turn may activate different defence response genes (see next section). Differences in the constitutive and induced expression levels in T- and S-oaks were observed for a number of transcription factors, including, e.g. several members of the *ERF* (ethylene-responsive factors) and *WRKY* transcription factor families (Figure 10).

Differences in the expression of genes related to DNA structure, especially histones, were highly apparent (Figure 10). Among the transcripts with higher constitutive expression levels in T-oaks compared to S-oaks, an enrichment of histone transcripts was obvious (Figure 2). For example, a transcript weakly similar to an *A. thaliana* histone 3B gene showed a distinctly higher level of expression in T-oaks (Tab. I). Functionally, DNA-associated histones may be involved in chromatin remodelling. Among the mechanisms of transcriptional regulation, chromatin remodelling accomplished through the activity of histone-modifying enzymes and ATP-dependent chromatin-remodelling complexes is emerging as a key process in the orchestration of plant biotic stress responses [39]. Whether the observed transcriptional differences in histones are related to differences in chromatin remodelling in T- and S-oaks remains an open question.

### Defence responses: metabolites

Metabolically, T-oak leaves were very rich in galloyl flavonol glycosides, condensed and hydrolysable tannins, and phenolic glycosides. Conversely, leaves of the S-oak genotype had a greater abundance of flavonoid glycosides and some related intermediates, of plastidic terpenoid intermediates, and of sugars and nucleotides. The results of our analysis are in agreement with those of our earlier investigation of soluble polyphenols performed with high-performance liquid chromatography (HPLC), which showed higher constitutive concentrations of some quercetin 3-glycosides and the PA precursor catechin in T-oaks than in S-oaks [29]. Moreover, the non-targeted metabolomic analysis revealed higher levels of plastidic terpenoid intermediates in S-oaks, which could be sustained and may therefore explain the increased herbivore-induced emission rates of monoterpenes previously observed in these lines [29].

#### Flavones and tannins

Flavonoids, particularly condensed tannins such as PA, are biologically active compounds that play an important role in plant-insect interaction [40,41]. The higher levels of galloylated flavonol glucosides in T-oaks compared to S-oaks after feeding (Figure 10B) may play a role in oak resistance. Once, it was believed that tannins were “quantitative defences” limiting protein digestion by herbivorous insects [42], but now it seems that the most important role of tannins is their pro-oxidant activity [43]. The oxidation of phenolics in the guts of insects produces ROS (e.g., quinones, peroxides), which can damage both essential nutrients and midgut tissues and therefore negatively influence insect performance [44].

The concentrations of hydrolysable and condensed tannins in *Quercus robur* have been considered to be negatively correlated with insect abundance since the first pioneering study [45]. Many other studies in different woody plant species have demonstrated the functional role of hydrolysable and condensed tannins as plant defences against generalist insects [46]. There are also counter-examples where generalists, such as the forest tent caterpillar, are sensitive to hydrolysable tannins, while others, such as the white-marked tussock moth (*Orgyia leucostigma*)[47] and the gypsy moth (*Lymantria dispar*)[13,43], are tannin-resistant. The same is true for condensed tannins [48]. The defensive effects of condensed tannins in *Quercus* sp. are even stronger on specialist insects [12]. For the autumnal moth (*Epirrita autumnata)*, it has been shown that high gallotannin concentration reduces the growth rate of this insect, whereas PAs impair larval growth only when the gallotannin content is low [49]. A more recent study [50] concluded that ellagitannins are the most bioactive tannins, while gallotannins have intermediate to low bioactivity and condensed tannins have low oxidative activities. Although we observed clear genotypic differences in tannin patterns and galloylated flavonol glycoside levels, the biological effect of these differences on green oak leaf roller larvae seem to be rather marginal. Our previous study [29,51] showed that larval mortality was equal on both oak genotypes, but larvae developing on T-oaks needed more leaf biomass to gain similar weights to larvae reared on S-oaks. Therefore, we speculate that the enrichment of gallotannins, such as corilagin, in the foliage of T-oaks may play a role in the defence properties in this resistant oak type. Furthermore, in our previous study, we found that some substances in S-oaks seem to attract female *T. viridana*[29]. In a recent study with a chrysomelid beetle, luteolin-7-glycoside was identified as a key substance in determining the attractiveness of plants to the females [52]. The beetles preferred the plants with high amounts of luteolin-7-glycosides [52]. This finding fits very well with our observation of high levels of luteolin-7-glycosides in S-oaks.

#### Terpenoids

In a previous study, we showed that *T. viridana* avoided T-oaks, which may have occurred because their blend of volatile organic compounds contained a higher ratio of sesquiterpenes and higher emission rates of α-farnesene and germacrene D [29]. The transcriptomic data (Figure 8) support the higher sesquiterpene activities and emission rates measured in T-oaks. We found higher expression levels (constitutive and induced) of a putative sesquiterpene synthase in T-oaks (Figure 5), which showed 75% amino acid identity to a germacrene D synthase of *Vitis vinifera* (XP_003634696.1).

We observed a clear increase in the transcript levels of plastidic MEP pathway-related genes and metabolites in S-oaks after pathogen feeding (Figure 8). Because the biosynthesis of monoterpenes originates in the plastidic MEP pathway [53], the observed transcriptomic and metabolomic data agree well with the more pronounced herbivory-induced emission of monoterpenes from the susceptible oak type observed in our previous study [29]. Herbivore feeding elicits the accumulation of traumatic acid (TA), as observed clearly in damaged oak leaves. This dicarboxylic acid is a potent wound-healing agent in plants that is associated with JA biosynthesis. The volatile form of JA, methyl jasmonate, induces the activation of sesquiterpene synthases [54]. Therefore, the different emission patterns of sesquiterpene (which is emitted at higher levels in T-oaks) and the nerolidol (a sesquiterpene) derivative DMNT (which is emitted at higher levels in S-oaks) observed in T- and S-oaks [29] might be under the control of the phytohormone JA, which was synthesised at different levels in these two lines (Figure 10B). However, further studies are needed to determine whether there is a direct involvement of JA in controlling the different terpenoid profiles of the T- and S-oaks.

### Different defence strategies of T- and S-oaks

The differences in the results of transcriptomic and metabolomic profiling between T- and S-oaks led us to the assumption that the two oak types follow different defence strategies. There are several theories about effective plant defence strategies against pathogens and herbivores. These include the Quantitative Defence Theory [55], the Optimal Defence Theory (ODT) [56,57], the Growth-Differentiation Balance (GDB) hypothesis [56,58,59], and the Resource Availability Hypothesis (RAH) [60-62]. Furthermore, there has been much general discussion of the advantages and disadvantages of constitutive and induced defence responses [63,64]; and references cited therein). However, the complexity of the defence response in plants often also leads to questioning of the proposed theories [55,57,63]. Herms and Mattson [58] got to the heart of all these theories with the title of their review “The dilemma of plants: To grow or defend”. This title highlights the recurring theme in all defence theories, which is that defence is costly.

The most important advantage to possessing constitutive defence, exemplified in T-oaks by the high levels of bioactive tannins, is the fast response during herbivore attack. These plants are defended from the first moment of attack, whereas a 'just-in-time’ induced defence strategy can take hours to days to protect the plant against such an attack. Furthermore, a constitutive defence can perhaps lead to a reduction in the number of attackers because, when volatile substances act as a direct defence, the insects may not even lay their eggs on the resistant plants. We suppose that this is the case for our T-oaks [29]. Our hypothesis that T-oaks follow a constitutive defence strategy is further supported by the higher levels of constitutive expression of sesquiterpenes and cell wall-degrading beta-glucanase genes.

Plants with induced resistance might have an advantage [63] if constitutive resistance against herbivores incurs fitness costs, such as reduced reproduction or lower growth rate. This advantage has been shown for the sticky monkey flower (*Diplacus aurantiacus*), where genotypes with higher resin concentrations had a lower growth rate [65]. Such allocation costs occur when large quantities of fitness-limiting resources are reserved for resistance traits. *Quercus robur* is one of the tree species that host the highest number of herbivorous insects [66]. Thus, most oaks are permanently attacked, but severe defoliation, e.g., by green oak leaf roller larvae, only occurs every five or six years and then only for one or two years in a very strong manner. It is therefore questionable whether the costs of these attacks justify a permanent resistance. The T-oak genotypes seem to follow the strategy of constitutive direct defence against the herbivores with the success to become less defoliated by the green oak leaf roller than S-oaks [29]. Interestingly, S-oaks have high constitutive transcript levels of a gene encoding the defence substance osmotin 34, which is known to function in the defence against biotic stress [67,68]. We do not know whether the specialist *T. viridana* is already adapted to a high level of osmotin34. However, the capability of forest insects to adapt to defence substances does exist, as demonstrated for the resistance of *Lymantria dispar* to tannins [13].

A commonly found trade-off between constitutive and induced defences occurs when the investment in constitutive defence is already high. In this case, fewer inducible defence responses exist [57,69]. This is exactly what we found for the two different oak types (Figure 1). In light of the present data, we conclude that the T- and S-oak types differ in their metabolic profiles and the levels of key metabolites and that T-oaks rather follow the strategy of constitutive defence, while S-oaks follow the 'just-in-time’ strategy of induced defence (Figure 10A, B).

## Conclusions

The transcriptomic and metabolomic differences identified in this study, together with our previous physiological and behavioural results, deepen our understanding of plant defence responses to specialist herbivore attack. Our data provide valuable information that paves the way for the identification of molecular and biochemical biomarkers. We identified several promising candidate genes in the functional groups 'protein’, 'secondary metabolism’, 'DNA’, and 'cell’. These sequences, together with transcripts of other functional groups, will be checked for SNPs and InDels that may explain the differences in defence between the two oak types. Additionally, further tests will be performed for the development of biochemical markers. The knowledge gained from this study provides the basis for developing a method for the early selection of potentially green oak leaf roller-resistant genotypes in natural pedunculate oak populations.

## Methods

### Plant and insect material

During an outbreak of *Tortrix viridana* L. (Lepidoptera, Tortricidae) in forest stands of North Rhine-Westphalia in 2003 to 2005, individuals of *Quercus robur* L. were classified as heavily defoliated and defined as susceptible ('S-oaks’) or were classified as only slightly defoliated and defined as resistant ('T-oaks’) using standard pictures to estimate the degree of defoliation [70]. These trees belonged to three genetically different populations (named 'Asbeck’, 'Muenster’ and 'Warendorf’) aged between 150 and 180 years (details on the oak stands were reported previously) [71]. We selected late and early bud-bursting individuals among both resistant and susceptible oaks. Among all forest stands analysed, the selected individuals of T- and S-oaks from the population 'Asbeck’ showed the most obvious differences in defoliation rate. In July 2008, 100 branches from eight individuals from the two tree groups were cut out the canopy and grafted onto *Q. robur* saplings to provide manageable oak material for our experiments [28].

Hybridisation between *Q. robur* and *Q. petraea* is quite common in natural oak populations, and the hybrids are often difficult to distinguish based on morphology [72]. Therefore, the selected individuals were tested for their species purity using eight microsatellite markers located in five different linkage groups [73]. Five of the eight grafted individuals were pure *Q. robur*. Thus, all experiments were carried out using these five pure clones of *Q. robur-*grafted plants (T-oaks: ASB2a, ASB14a, ASB17a; S-oaks: ASB13b, ASB47b). More detailed information about these oak clones and the rearing of the insects has been given previously [29].

### Preparation of the oak material for RNA analysis

At the end of April 2009, one 3^rd^ or 4^th^ instar larva of *T. viridana* was placed on each of 10 totally unfed grafted oaks per clone (the above-mentioned three T-oak clones and two S-oak clones). The experiment was performed within a phytochamber with the light switched on during the 16 h the experiment lasted. These 50 trees and 50 additional oaks without larvae (uninfected control plants) were covered with gauze to prevent larvae from breaking out and, for the control plants, to have the same experimental conditions. After 16 h of rearing, the larvae were removed and both fed and unfed leaves from treated (FED) and control plants (CO) were individually frozen in liquid nitrogen immediately after the experiment.

Because the budburst of the five clones differed slightly, the experiment was performed during a time span of 14 days, so the leaves used for the experiments were at the same developmental stage for all clones.

### RNA isolation

Because of the high levels of phenolic compounds in oak leaves, which are known to hamper RNA extraction, a method based on the protocol originally published by Boom *et al.*[74] and modified by Hahn [75] was used. The only further modification was storage of the RNA at -70°C instead of -20°C.

### RNAseq analysis

For the T-oak fed sample, RNA was prepared from three clones with three individuals per clone. For the S-oak fed sample, RNA was prepared from two clones with three individuals each. The RNA samples were pooled for each tree sample and used for sequencing. Two separate cDNA libraries were created from 1 μg RNA of each of the two samples by oligo-dT priming (GATC Biotech AG, Konstanz, Germany). Both libraries were sequenced by GATC Biotech AG (Konstanz, Germany) using an Illumina/Solexa Genome Analyser to create single-end reads of 36 bp length (12.5 million reads for T-oaks and 12.3 million reads for S-oaks; Solexa reads available at the Short Read Archive (SRA) at EMBL-EBI [EMBL: ERP002577]). Sequencing of unfed control plants was performed using the two above mentioned T-oak clones and two of the above-mentioned S-oak clones with 1 and 2 individuals per clone, respectively. Two separate cDNA libraries were created from 1 μg RNA and sequenced by GATC Biotech AG (Konstanz, Germany) using an Illumina/Solexa Genome Analyser to create single-end reads of 101 bp length (80.5 million reads for T-oaks and 124.8 million reads for S-oaks; Solexa reads available at the SRA at EMBL-EBI [EMBL: ERP002577]).

### Bioinformatic analyses of the RNAseq data

#### Generation and annotation of a Q. robur reference set of transcript sequences

For *Q. robur*, no genomic sequence is available. Therefore, a nearly non-redundant *Q. robur* reference set of transcript sequences (*Q. robur* reference set) was created *in silico* for the subsequent quantification of the sample-specific transcripts. The reference set consisted of 7,170 *Q. robur* Unigene sequences (NCBI, v1) and 7,377 additional *Q. robur* ESTs from Evoltree [76,77]. All corresponding reference sequences (14,547 sequences) were annotated using the MapMan ontology which is specifically tailored to plants and has been designed to be as free of redundancy as possible [30]. The sequences were assigned to MapMan BINs (functional classes/subclasses) and specific gene functions were predicted using the Mercator tool [78]. The prediction of gene function by Mercator is based on similarity to known plant sequences, especially to *A. thaliana,* and to conserved protein domains. More than 52% of the reference transcripts were annotated in MapMan (Additional file 6).

#### Transcript quantification in the four Q. robur samples

Transcripts were quantified in each of the four pooled samples by mapping the related trimmed reads to the 14,547 sequences of the *Q. robur* reference set using the Read Mapper (Beta v1.0 program of the CLC Genomics Workbench 5.1 suite; CLC bio, Aarhus, Denmark) with default parameters (but with 0.9 overlap and 0.95 identity). Nonspecific matches were randomly treated by default. As an expression measure, RPKM was used in an effort to normalise for the differences in the numbers of mapped reads between the different samples. Approximately 35% of the reads from the control samples and approximately 53% of the reads from the fed samples mapped to the reference set (data not shown). The derived RPKM values of each reference gene are summarised for all four samples in Additional file 6.

Log2 fold changes for the expression values (RPKM values) from the following sample comparisons are listed in Additional file 6: T-oak control (T_CO_) versus S-oak control (S_CO_), T-oak fed (T_FED_) versus S-oak fed (S_FED_), T-oak fed versus T-oak control (T_IND_), and S-oak fed versus S-oak control (S_IND_).

Only those transcripts showing any value when deriving log2 fold changes (i.e., transcripts with RPKM values different from zero) in each of the compared samples were included in the subsequent analyses to avoid incorrect results due to a missing representation of a transcript in one sample caused by variation in the library preparation or the sequencing procedure.

#### Transcript mapping to MapMan BINs and different MapMan pathways, and Wilcoxon Rank sum test of BINs

For each of the analysed sample comparisons, transcript identifiers and the related log2 fold ratios were imported into the MapMan desktop tool [30] (v3.5.1.; downloaded from MapMan Site of Analysis) [79]. In addition, the MapMan annotation file for the *Q. robur* reference set (see above) was imported into the tool. Thus, data were mapped to MapMan BINs, which allowed the visualisation of the data on different MapMan pathways and other biological processes.

Using the Wilcoxon rank sum test integrated in the MapMan tool, BINs were identified that showed an average BIN response that was significantly different from the response of the other BINs, as indicated by their corrected p-values in the test (Benjamini Hochberg correction; False Discovery Rate (FDR) < 5%, p < 0.05) [80].

#### Selection of specific transcript groups (Groups of differentially expressed transcripts and induced transcripts)

To compare the transcript levels of T- and S-oaks after feeding, all transcripts with log2 fold changes ≥ 1.5 (T_FED_ > S_FED_) or ≤ -1.5 (T_FED_ < S_FED_) were selected as transcripts that were differentially expressed between T- and S-oaks after feeding (Additional file 3). All transcripts with log2 fold changes ≥ 1.5 (T_CO_ > S_CO_) or ≤ -1.5 (T_CO_ < S_CO_) were selected as transcripts that were differentially expressed between T- and S-oak controls (Additional file 3).

To identify transcript changes induced by *T. viridana* feeding in T- or S-oaks, all transcripts with T_IND_ (T_IND_ = log_2_(T_FED_/T_CO_)) values and S_IND_-values of ≥ 1.5 or of ≤ -1.5 were selected as transcripts induced by *T. viridana* feeding in both T- and S-oaks. Up-regulated transcripts showed log2 fold changes ≥ 1.5, while down-regulated transcripts showed log fold changes ≤ -1.5 (Additional file 3).

#### Analysis of functional over- and under-representation

Over- and under-representation analysis of MapMan BINs in different transcript groups was carried out using the plugin BiNGO [81] (v2.3) for the software package Cytoscape [82] (v2.6.1). A MapMan ontology file was created for BiNGO using a PERL script. The *Q. robur* reference set with the assigned MapMan annotation (see above) was used as a reference for the over- and under-representation analysis. A related *Q. robur* MapMan annotation file was created for BiNGO using a PERL script. Statistically significant BINs consisting of either over- or under-represented transcripts were selected according to their corrected p-value (False Discovery Rate, FDR rate ≤ 2%) using a hypergeometric test.

### cDNA synthesis and semi-quantitative PCR

For semi-quantitative PCR experiments, RNA was isolated from the five oak clones as described previously, and cDNA was synthesised by oligo-dT priming based on the SMART PCR cDNA Synthesis KIT (Clontech Laboratories, USA; Protocol No. PT3041-1).

For validation of the expression value results for candidate genes by semi-quantitative PCR, cDNAs were pooled from the same number of individuals per clone as for the RNAseq analysis. Following a standard protocol, PCR reactions contained appropriate amounts of template cDNA (2 to 10 ng), 50 mM KCl, 20 mM Tris–HCl (pH 8.4), 1.8 mM MgCl_2_, 200 μM dNTPs, 1 unit Taq polymerase, and 0.4 μM of each primer (detailed primer information is given in Additional file 7) in a total volume of 25 μl. PCR was carried out in a Biometra Personal Thermocycler (Göttingen, Germany) with a pre-denaturation step at 94°C for 4 min, followed by 25 cycles of 93°C for 1 min, incubation at a suitable annealing temperature for each primer combination (50°C to 60°C) for 45 sec, and 72°C for 1 min, followed by a final elongation at 72°C for 5 min. PCR amplification products were checked on a 1.2% agarose gel in 0.5 x TBE buffer stained with RotiSafe (Carl Roth GmbH + Co. KG, Karlsruhe, Germany). SmartLadder (Eurogentec, Cologne, Germany) was used as the size standard.

PCR was conducted with (i) different cycle numbers (25, 30 and 32) and (ii) different template cDNA concentrations to validate the linearity of the measured expression values.

### Description of the material for the metabolomic analyses

Metabolomic analysis was performed from the same leaf material as used for RNAseq. In addition, all leaf material collected for the physiological and behavioural experiments described in Ghirardo *et al.*[29] was analysed covering metabolomic changes 32 h after onset of insect feeding. Details of materials and methods can be found in Ghirardo *et al.*[29]. In brief, plants were fed by 3^rd^ or 4^th^ instars of *T. viridana* under controlled conditions inside a phytochamber (16/8 h light/darkness). Shoots of T- and S-oaks were separately enclosed into Perspex glass cuvettes and grown for 48 h (16 h unfed followed by 32 h feeding) at 19°C and 50–150 μmol photons m^-2^ s^-1^ PAR (bottom-top). Harvested leaves of fed plants were separated between (i) T-oaks (“T” leaves) and S-oaks (“S” leaves), (ii) leaves, directly damaged by larvae (“D” leaves) and intact (“I” leaves; untouched leaves randomly selected - 4 leaves for each plant - of the same fed plants), (iii) plants with a leaf stage of development that naturally experience the larvae feeding; i.e. 2–4 weeks after bud break (“Y” (young) leaves) and plants start to host the oviposition process of adult female moth of *T. viridana;* i.e. 6–8 weeks after bud break (“O” (old) leaves). Individual experiments were performed with 4 different clones (T-oaks: ASB17a, ASB2a; S-oaks: ASB47b, ASB13b) and 4–5 biological replicates for each clone.

### Non-targeted metabolomics

Non-targeted metabolome analysis was achieved by molecular mass assignment of high-resolution mass spectra obtained using a Fourier Transform Ion Cyclotron Resonance Mass Spectrometer (FT-ICR-MS, APEX Qe, Bruker, Bremen, Germany) equipped with a 12-Tesla superconducting magnet and an Apollo II electrospray (ESI) source.

Metabolites were extracted from 20 mg of each sample with 500 μL CH_3_OH:H_2_O solution (1:1, v:v) for 15 min in ultrasonic bath. After centrifuging for 10 min. at 10,000 rpm, 400 μL of supernatant was further diluted with 500 μL of CH_3_OH:H_2_O (75:25, v:v). Samples were kept at 4°C and introduced at a flow rate of 2 μL min^-1^ into the ionization source (ESI), run in negative operation mode and therefore generating mono-charged ions. The spectra were acquired with a mass-to-charge ratio (m/z) range of 120–1,000 and a time domain of 1 Megaword. Spectra were internally calibrated using both primary and secondary metabolites; calibration errors were always below 0.05 ppm. Peak lists were obtained exporting peak mass intensities of FT-ICR ESI (-) spectra with a signal to noise (S/N) ratio of two. Peak lists of different samples were aligned into a single matrix within a precision of > 0.7 ppm.

### Analysis of the metabolomic data

Data were analysed using a multivariate data analysis (MDA) approach using the software package 'The Unscrambler’ (v. 8.0, CAMO A/S, Norway). First, data were analysed by PCA, using the peak list as X-variable, logarithmically transformed with X = log2X. The PCA was calculated after centering the data and weighting the data with 1 s.d.^-1^ (unit variance). Significant discriminant masses between T- and S-oaks (T vs. S), systemic and local responses (I vs. D), and developmentally different leaves (O vs. Y) were searched by partial least square regression (PLSR) and Martens’ test [83]. In the PLSR, Y-values described either the genotype, (with T = 1 and S = 0), or the systemic responses (with D = 1 and I = 0), or the age of the leaves (with O = 1 and Y = 0) and the X-values contained the matrix of mass intensities with a threshold of 6.37e^5^. For identification of significant discriminant masses, annotation was automatically achieved via the portal MassTRIX3 [84,85], by using KEGG/API [86]. For the annotation we used KEGG combined with Human Metabolome Database (HMDB) [87] and with expanded lipids from LipidMaps (LMPK; version 06–2011: As reference organism we selected *Populus trichocarpa* because species of the genus *Quercus* are not included so far) [88]*.* In addition, the structure of uncertain annotated metabolites was confirmed with ChemSpider [89]. Next, the results were filtered manually with a maximal mass error acceptance of 1.3 ppm, the error caused by spectra alignment. Finally log2 ratios of mass spectra intensities were calculated for T/S, O/Y, D/I in order to visualise in HeatMaps up- or down- regulation of the different metabolites grouped into the main metabolic pathway according to KEGG [31].

### Mapping of transcriptomic and metabolomic data to KEGG pathways

The web-based functional annotator KAAS (KEGG Automated Annotation Server) [51,90] was used to map the transcript identifiers to KO numbers thus assigning the transcripts to KEGG pathways (single bidirectional best hit-method using the representative set for genes at KAAS; default blast score of 60). All metabolites were included in the pathway mapping, which showed statistically significant discriminant masses between T- vs. S-oaks after feeding (see above). The selected masses were mapped to specific metabolites in KEGG pathway displays using MassTRIX3 [84,85]. Log2 fold ratios of mapped transcripts and metabolites were displayed onto the KEGG pathways in color code.

## Abbreviations

BINs: MapMan functional categories; CDPK: Calcium-dependent kinases; DMNT: Homoterpene 4,8-dimethylnona-1,3,7-triene; FA: Fatty acids; FT-ICR-MS: Fourier Transform Ion Cyclotron Resonance Mass Spectrometer; GDB: Growth-Differentiation Balance hypothesis; HIPVs: Herbivory-induced plant volatiles; HPLC: High-performance liquid chromatography; JA: Jasmonic acid; MDA: Multivariate data analysis; MAPK: Mitogen-activated protein kinase; MEP pathway: 2C-methyl-D-erythritol 4-phosphate pathway; ODT: Optimal Defence Theory; PA: Proanthocyanidins; PCA: Principal components analysis; PLSR: Partial least squares regression; RAH: Resource Availability Hypothesis; RNA-seq: RNA sequencing; ROS: Reactive oxygen species; RPKM-value: Reads per kilobase of exon model per Million mapped reads; S-oak: Susceptible oak type; TA: Traumatic acid; T-oak: Resistant oak type

## Competing interests

The authors declare that they have no competing interests.

## Authors’ contributions

BK performed the bioinformatic analysis, the combined mapping of transcriptomic and metabolomic data, and drafted the manuscript. AG contributed to the experimental design, processed the metabolomic data, performed MDA analysis and participated in drafting the manuscript. JPS participated in conceiving of the study, its design and coordination, contributed to the metabolomic analysis, and participated in drafting of the manuscript. BaK and PSK contributed by running the non-targeted metabolome analysis. MF participated in conceiving of the study, its design and coordination, and participated in drafting of the manuscript. HS conceived of the study, designed and coordinated it, performed and analysed gene expression data and drafted the manuscript. All authors read and approved the final manuscript.

## Supplementary Material

Additional file 1 **MapMan BINs with significantly different transcriptional overall response in T-oaks compared to S-oaks.** MapMan BINs with significantly different average BIN responses compared to the response of all other BINs (p < 0.05, Wilcoxon rank sum test in the MapMan tool) are listed for the different sample comparisons. Click here for file

Additional file 2 **Most significant MapMan BINs with different transcriptional overall response in T-oaks compared to S-oaks.** MapMan BINs with most significantly different average BIN responses compared to the response of all other BINs (p < 0.025, Wilcoxon rank sum test in the MapMan tool; Additional file 6) are presented for the different sample comparisons. Click here for file

Additional file 3 **Candidate gene groups with related raw data of transcript quantification.** Functional annotation (MapMan), transcript counts and RPKM values in the different samples as well as derived log fold changes of RPKM values (for the indicated sample comparisons) are summarized for different candidate gene groups. “T and S_ind_up”, T_IND_ > = 1.5 and S_IND_ > = 1.5); “T and S_ind_down”, T_IND_ ≤ -1.5 and S_IND_ ≤ -1.5). T_FED_ > S_FED_/T_CO_ > S_CO_, log2 fold changes ≥ 1.5; T_FED_ < S_FED_/T_CO_ < S_CO_, log2 fold changes ≤ -1.5. Click here for file

Additional file 4 **Nucleotide sequences of the transcripts listed in Tables** 1**, **2**, and**3**.** All nucleotide sequences assigned to the transcripts identifiers which are listed in Tables 1, 2, and 3 are provided. Click here for file

Additional file 5 **Complete list of annotated masses for metabolomic identification.** Compounds are sorted according to which KEGG [31] group the compound belongs (alphabetically ordered) and then by values of log2 T/S ratios (increasing). All log ratios displayed are significant (Martens’ test [83]; otherwise value set to 0). In bold are highlighted the compounds reported in results section. Columns: (A) compound number; (B) exact annotated mass; (C) the error in ppm; (D), the ID of KEGG/HMDB/LMGP/ChemSpider databases [31,88,89]; (E), the molecular formula (obtained from KEGG) [31]; (F) the proposed identification with possible isomers, (G) the additional (marked with “x”) identification of the corresponding C^13^ and (H) O^18^ isotopes; (I) category of which averages (AV) and standard error (S.E.) of MS intensities refer to; (J) the averages and (K) S.E. of MS intensities of resistant (T; n = 26) or old (O; n = 16) or damaged (D; n = 26) leaves and (L) averages and (M) S.E. of MS intensities of susceptible (S; n = 26) or young (Y; n = 38) or intact (I; n = 28) leaves; (N) log2 ratios of T/S, (O) log2 ratios of O/Y; (P) log2 ratios of D/I; (Q) main KEGG compound group, (R-U) alternative pathways for isomers or compounds belonging to more groups. Click here for file

Additional file 6 **Raw data of transcript quantification for all transcripts of the reference set in the analysed samples.** Functional annotation (MapMan), transcript counts and RPKM values in the different samples as well as derived log fold changes of RPKM values (for the indicated sample comparisons) are summarised for all transcripts (Identifiers) of the reference transcript set used for mapping the Solexa reads. Click here for file

Additional file 7 **Detailed information of primers used for validation of the candidate genes.** The associated gene, names of primers, primer sequences and the used annealing temperature are given. Within the primer names F means the forward and R means the reverse primer. Click here for file
