# Supplementary figures and images for: Integrated transcriptomics and metabolomics decipher differences in the resistance of pedunculate oak to the herbivore Tortrix viridana L
Source: BMC Genomics. 2013 Oct 28;14:737. doi: 10.1186/1471-2164-14-737 (PMC4007517; doi:10.1186/1471-2164-14-737)

## Slide 1
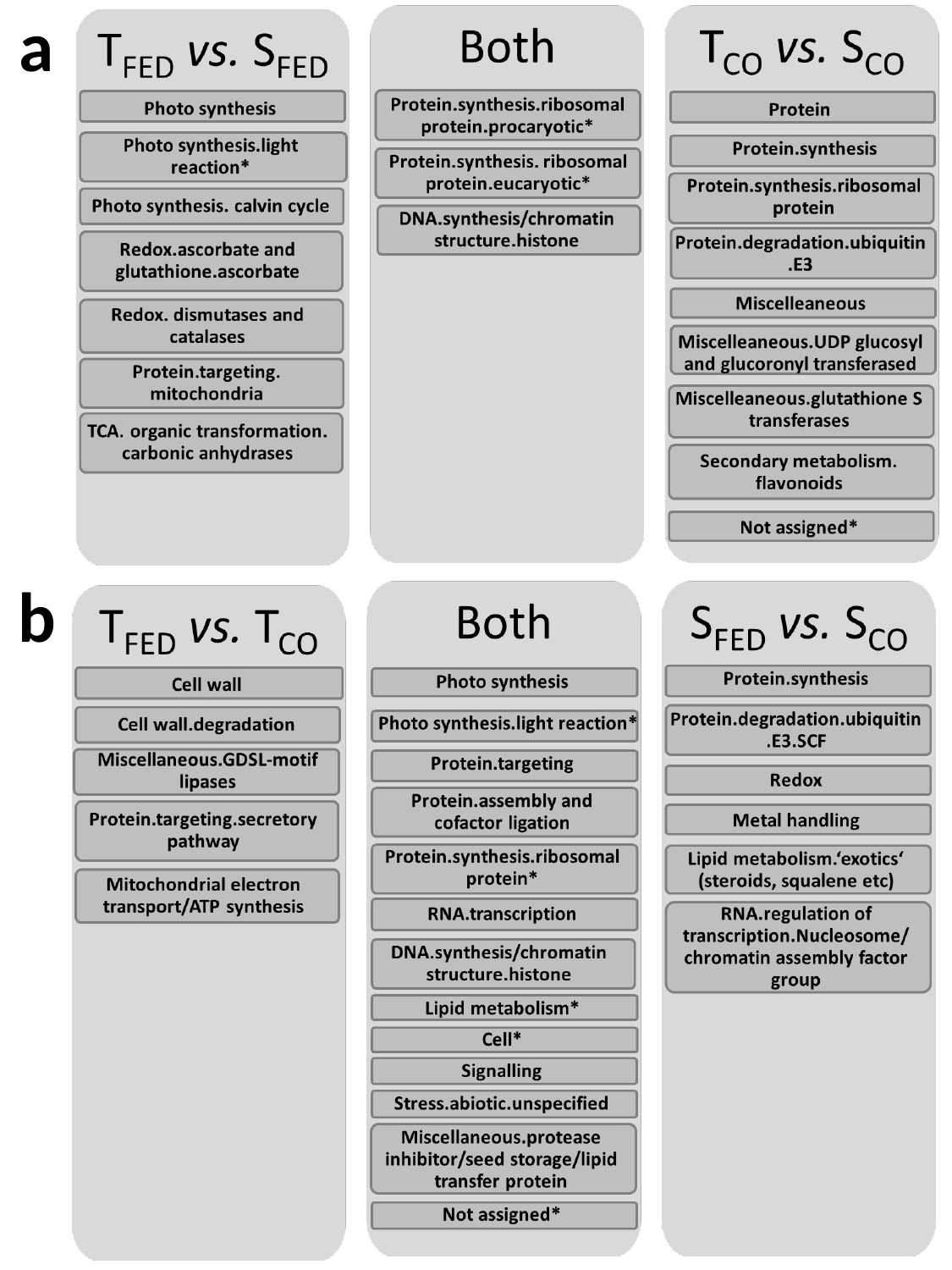

a
b

Supplement: Additional file 2 — Most significant MapMan BINs with different transcriptional overall response in T-oaks compared to S-oaks. MapMan BINs with most significantly different average BIN responses compared to the response of all other BINs (p < 0.025, Wilcoxon rank sum test in the MapMan tool; Additional file 6) are presented for the different sample comparisons. [file 1471-2164-14-737-S2.pptx]
